# DON6D: a decoupled one-stage network for 6D pose estimation

**DOI:** 10.1038/s41598-024-59152-x

**Published:** 2024-04-10

**Authors:** Zheng Wang, Hangyao Tu, Yutong Qian, Yanwei Zhao

**Affiliations:** 1 School of Computer and Computational Sciences, Hangzhou City University, Hangzhou, 310015 China; 2 School of Computer Science and Technology, Zhejiang University of Technology, Hangzhou, 310023 China; 3 School of Engineering, Hangzhou City University, Hangzhou, 310015 China

**Keywords:** 6D pose estimation, Deep learning, Real-time method, Mathematics and computing, Computer science

## Abstract

The six-dimensional (6D) pose object estimation is a key task in robotic manipulation and grasping scenes. Many existing two-stage solutions with a slow inference speed require extra refinement to handle the challenges of variations in lighting, sensor noise, object occlusion, and truncation. To address these challenges, this work proposes a decoupled one-stage network (DON6D) model for 6D pose estimation that improves inference speed on the premise of maintaining accuracy. Particularly, since the RGB images are aligned with the RGB-D images, the proposed DON6D first uses a two-dimensional detection network to locate the interested objects in RGB-D images. Then, a module of feature extraction and fusion is used to extract color and geometric features fully. Further, dual data augmentation is performed to enhance the generalization ability of the proposed model. Finally, the features are fused, and an attention residual encoder–decoder, which can improve the pose estimation performance to obtain an accurate 6D pose, is introduced. The proposed DON6D model is evaluated on the LINEMOD and YCB-Video datasets. The results demonstrate that the proposed DON6D is superior to several state-of-the-art methods regarding the ADD(-S) and ADD(-S) AUC metrics.

## Introduction

In recent years, with the development of the robotic industry, related industrial applications have been widely deployed. The six-dimensional (6D) object pose estimation is one of the important tasks in the field of robotics, and it can be used in a variety of important scenarios, such as robotic grasping^1,2^ and autonomous driving^3^.

The 6D pose estimation is challenging to tackle due to variations in lighting, sensor noise, occlusion of scenes, and truncation of objects. The 6D pose estimation methods of a target object provide a robot with abundant information on the two-dimensional (2D)-three-dimensional (3D) spatial interactions^4^. However, the 6D posture, which includes the translation transformation of three degrees of freedom and the rotation transformation of three degrees of freedom, is often considered a coordinate transformation obtained from the object coordinate system to a camera coordinate system.

The existing works^5,7^ leverage the advantage of two-stage methods of a pose refinement module that is added at the end of the model to obtain more precise pose prediction results. The pose refinement module usually applies Perspective-n-Point (PnP) or Iterative Closest Point (ICP) to transform the pose matrix from the camera coordinate system to the object coordinate system. However, this module is very time-consuming, and these methods are typically trained using a surrogate target^8^ and adopt a 2D error loss function, which results in a relationship between the errors and the pose prediction accuracy that is not a one-to-one relationship^13^. Therefore, the result does not reflect the true object of the pose estimation. However, some studies^13–15^ applied a one-stage method and removed the pose refinement module, replacing it with a learnable network module, which increased the learnability of the network. Nevertheless, Cheng et al.^15^ proposed a method of intra- and inter-modality fusion for 6D pose estimation. However, this method is poorly interpretable due to the complex fusion and coupled pose estimation. The learnable module used to solve the final pose matrix in the work of Hu et al.^13^ is coarse, resulting in a lack of accuracy. At the same time, too many parameters can be easily produced when integrating pixel-wise features at various scales^16^, which can decrease the inference speed. In conclusion, the one-stage methods can compensate for some of the shortcomings of the two-stage methods, but they still suffer from poor interpretability and unbalanced accuracy and speed gains.

**Figure 1:**
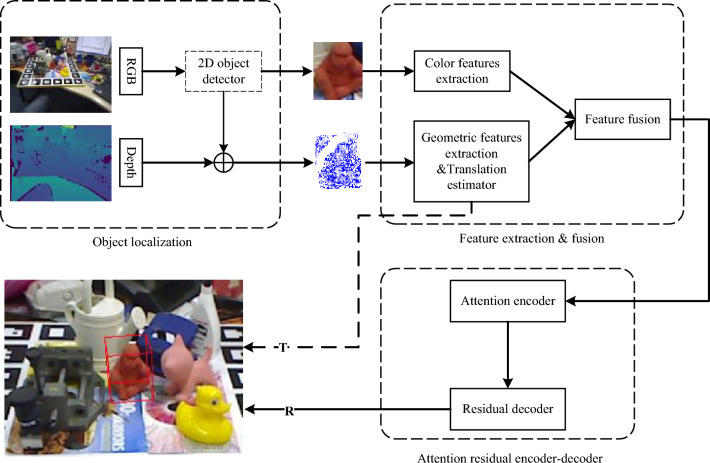
Flowchart of the proposed DON6D model. The DON6D model contains the object localization module, feature extraction and fusion module with a dual data augmentation function, ARED with an attention mechanism, feature-key point vector pipe, and box residual pipe.

In view of the aforementioned, this work proposes a decoupled one-stage method (DON6D) network model for 6D pose estimation to overcome the existing problems related to low accuracy, interpretability, and speed. The flowchart of the proposed model is shown in Fig. 1, where it can be seen that it uses an attention residual encoder–decoder (ARED) to accelerate the object pose prediction using fully fused features as input data. Due to the inconsistency in the prediction process of the rotation and translation matrices, a decoupled approach is used in the proposed model to allocate computational resources, which enhances the network`s interpretability. Lacking diversity in training data can result in model overfitting in training, so the proposed model adopts a dual data augmentation (DDA) strategy to address this problem. In contrast to the other approaches based on rendering backgrounds and composing objects, the DDA is a low-cost but effective method. The feature fusion block is fast, and an attention encoder (AE) is added to compensate for the possibility of the loss of accuracy. The attention mechanism of the encoder enables the network to attend to regions of interest efficiently to reduce the number of model parameters. This is an effective strategy to improve the inference speed, together with a residual decoder (RD), which follows the attention mechanism. The RD is differentiable and trainable, which replaces the refinement modules^5,7^; it is an effective module that considers both speed and precision.

The proposed method is evaluated on two datasets, the LINEMOD and YCB-Video datasets. The experimental results demonstrate that the proposed method can outperform the state-of-the-art (SOTA) methods for most types of objects.

To summarize, the main contributions of this paper are as follows:

- An effective dual data augmentation (DDA) strategy that overcomes the lack of diversity in training data in the feature extraction module is proposed. This strategy does not introduce additional rendering and synthesis costs;
- The AE that can make the network focus on key feature regions is presented;
- The RD is proposed to replace the traditional refinement module, which improves the speed of network inference and ensures accuracy.

## Related work

### Pose estimation using two-stage methods

Some previous works have used two-stage methods, which add a pose refinement module at the model end. These methods usually first extract 2D features from an image and use the PnP^19^ or ICP^17^ module at the end. The PoseCNN^17^ allows the decoupling of 6D pose estimation for small or symmetric objects, and it has been the first and most influential 6D pose estimation network that applies the ICP to the pose prediction. However, it is slow due to pose refinement. A segmentation-driven method^18^ can easily lose the correct 3D bounding box when an object is obscured, which can affect the calculation accuracy of the PnP. The PVNet^7^ predicts the direction of each pixel to each key point and allows uncertainty-driven PnP to measure the 6D pose. The segmentation-driven method^18^ and PVNet^7^ also perform segmentation and voting for each correspondence to increase robustness. In addition, to compensate for the low speed of some two-stage approaches, a number of algorithms^6,11^ use only RGB images as input data. In^5,8^, the authors exploited special pose refinements that are differentiable and trainable.

### Pose estimation using one-stage methods

One-stage methods represent end-to-end architectures and are usually faster than the two-stage methods. The G2L-Net^14^ extracts features from point clouds and then segments the point cloud to narrow the solution search space. At the same time, it uses the point-wise embedding vector features and rotation residual estimator to accelerate the inferencing process while improving estimation accuracy. The methods proposed in^15,20,21^ use a semantic segmentation network to narrow the solution search space. In general, a semantic segmentation head is slower in locating than an object detection head^14,22^. The single-stage approach^13^ integrates the RANSAC-based PnP algorithm into the network to construct an end-to-end network. That is a one-stage method to improve estimation accuracy and speed. Most current methods^16,23^ provide innovative concepts for one-stage networks. The CloudAEE^23^ exploits only point clouds for training to reduce the overhead of synthesizing color images. In^16^, it has been shown that using a multiscale pixel-wise feature fusion module instead of refinement is effective in improving the accuracy of object recognition.

## Proposed method

### Overview

**Figure 2:**
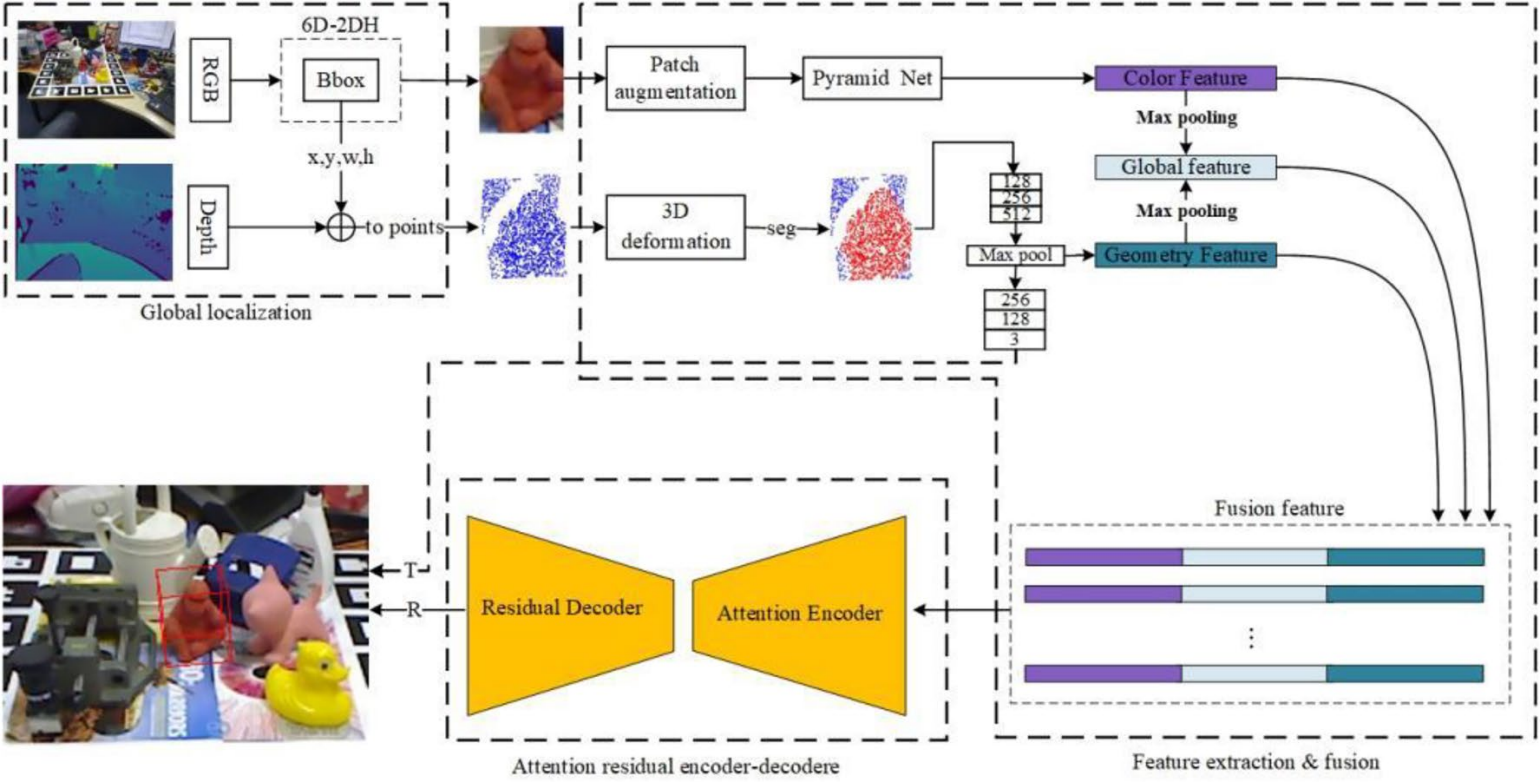
DON6D framework. For the given RGB image and depth image, the proposed DON6D adopts the 6D-2DH to locate the object as input. Then, the RGB image patch and the point cloud patch of the object are fed to the color pipe and the geometry pipe to extract data features, respectively. The term "seg" in the figure refers to the segmentation procedure of the object and background point clouds. In the geometry pipe, the translation matrix of the object is outputted at last. After fusing the features, the DON6D applies the ARED to estimate the rotation matrix.

In this section, the proposed DON6D network is described in detail, and its framework is shown in Fig. 2. The proposed framework first detects the object location based on the RGB-D input data. Then, the detected color information and point cloud information, obtained from a depth image, are fed to different feature extractors. More specifically, a Pyramid network is used to extract color features from an RGB image that has undergone patch augmentation, and an improved PointNet^24^ is employed to extract geometric features from point clouds that have undergone minor 3D deformations^22^. While extracting the geometric features, object translation is performed, and after that, these features are concatenated in the channel dimension. Finally, the ARED is applied to obtain the final 6D pose.

### Object localization

According to the previous work^5^^,^^25^, learning the 6D pose of objects to be approximated from RGB-D images directly is challenging. Therefore, first, it is necessary to limit the 3D search space maximally to extract color and geometric features individually while preserving the intrinsic structure of data sources. Unlike the semantic segmentation algorithms used in^5^^,^^16^^,^^25^, the proposed method requires only the bounding box of an object and, thus, can locate the object faster than the existing methods. In this work, a fast 2D object detector called the 6D-2DH (i.e., the 2D detection head for 6D pose estimation) is used to locate an object`s position in RGB-D images.

**Figure 3:**
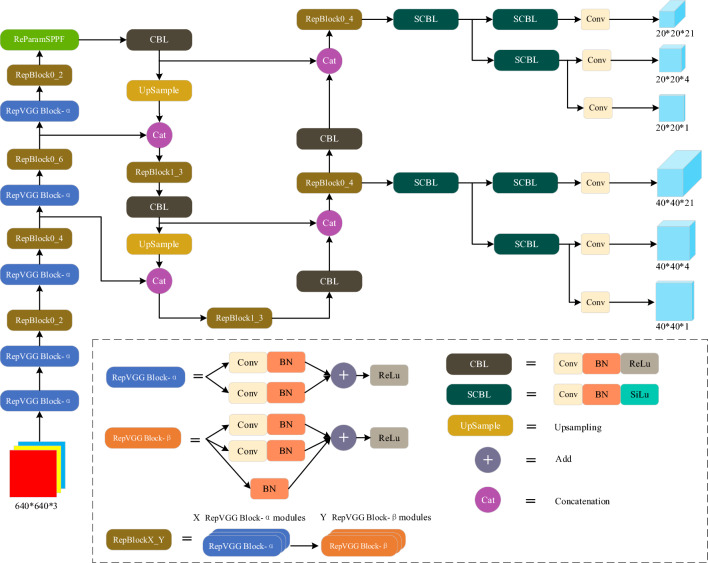
6D-2DH structure.

The network structure of the 6D-2DH contains three main sections: the backbone section, the neck section, and the detection head section, as shown in Fig. 3. Inspired by the work of Li et al.^26^, the 6D-2DH extends its design in the neck section by introducing certain improvements to the backbone and detection head sections. In summary, this section focuses on designing a simple object detection network using a re-parameters spatial pyramid pooling – Fast (RP-SPPF).

Spatial pyramidal pooling (SPP) was first proposed by He et al.^27^, and it can convert feature maps of arbitrary size into feature vectors of a fixed size. The SPP mainly solves the problem of image distortion caused by performing the cropping and scaling operations on image regions, as well as the problem of repeated extraction of relevant features from images by convolutional neural networks. By adopting the SPP operation, the candidate frame generation of the model becomes much faster, and the computational cost can be reduced. Based on the SPP, Glenn^28^ proposed a faster spatial pyramidal pooling (SPPF) model. In general, the prediction accuracy of this model can be improved by increasing the number of parameters, but at the same time, the increase in the number of parameters can reduce the model inference speed. Li^26^ and other researchers used the ReLu function to replace the activation function in the SPPF to improve the inference speed, but this still did not bring any substantial changes to the spatial pyramid pooling structure.

**Figure 4:**
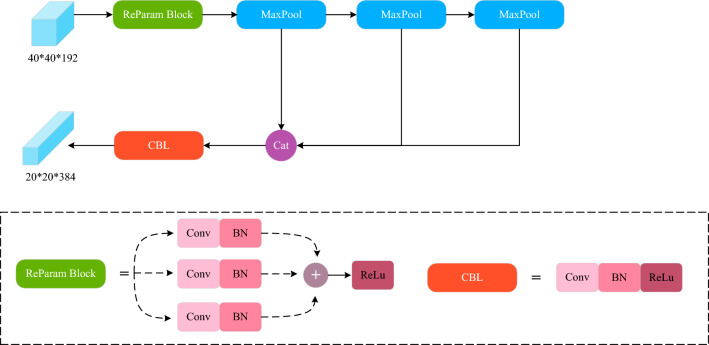
RP-SPPF structure.

The idea of parametrization has resolved the conflict between the number of parameters and the inference speed to a certain extent. First proposed by Ding et al.^29^, the main idea of re-parameters is that the model training structure corresponds to a set of training parameters, and then another inference structure is used in the inference phase, and the training parameters are equivalently converted to inference parameters. This allows the model to use a large overhead during training while using a small overhead during the inference phase. As the convolution is linear (i.e., the convolution possesses additivity), by combining the idea of re-parameters with the SPPF model structure, the RP-SPPF model is designed and applied to the 6D-2DH model, as shown in Fig. 4.

The 40 × 40 × 192 feature map is fed to the RP-SPPF module and processed by a re-param block structure and three identical max-pooling layers to extract important information from the features. Further, the three resulting feature maps are merged in the channel dimension, as shown in Fig. 4. Finally, the stitched features are processed by a convolutional block CBL. As this image dataset contains a large number of duplicates, redundant blocks of background pixels are implemented in addition to the edge information and color information on the target object. Therefore, after extracting the important features using the max-pooling layer, they are not combined with the original input feature map.

### Feature extraction and fusion

The RGB images contain visual information about low-textured objects, high occlusion, and various lighting situations; meanwhile, the depth images offer additional geometric information. During feature extraction and fusion, the main challenge is how to extract relevant color and geometric features and fuse them. The physical significance and distribution information of these features exist in separate spaces^5^, ^10^, despite the fact that they have comparable storage formats. In addition, experiments have shown that the training data of the same object are highly similar, which can significantly affect the model performance on the validation and test sets. To enhance the generalization ability, performing data augmentation on training data is necessary; this will be verified in the subsequent ablation experiments. To this end, this study proposes a feature extraction-fusion mechanism with the DDA function. In addition, two pipes, namely the color pipe and geometry pipe, are used to extract color and geometric features, respectively.

#### Color pipe

In general, occlusion problems can make the 3D object pose estimation in realistic scenarios challenging, which can further make the ground truth of objects difficult to predict because their visual features are hidden. This study uses a data augmentation method named patch augmentation, which was proposed in^6,8^, to simulate truncation after acquiring an RGB image *I*_*rgb*_ of the target object recognized by the 6D-2DH. Unlike in^6,8^, this study dynamically intercepts a fixed-length patch image from *I*_*rgb*_. A patch image is defined as follows:

1 $$ \left\{ \begin{gathered} \mu_{x,y} = \frac{{\left( {w,h} \right) - s}}{2} \hfill \\ \sigma_{x,y} = \frac{{(w,h) - s - \mu_{x,y} }}{3} \hfill \\ {\text{x}},y = \phi (\mu_{x,y} ,\sigma_{x,y} ) \hfill \\ \end{gathered} \right., $$

where *w* and *h* are the width and height of *I*_*rgb*_, respectively; *μ*_*x,y*_ is the mean; *σ*_*x,y*_ is the variance; *ϕ* represents a random set of *x*,*y* drawn from a normal distribution; *x*,*y* are the coordinates of the upper left corner of a patch image in *I*_*rgb*_; *s* is the size of a patch image.

**Figure 5:**
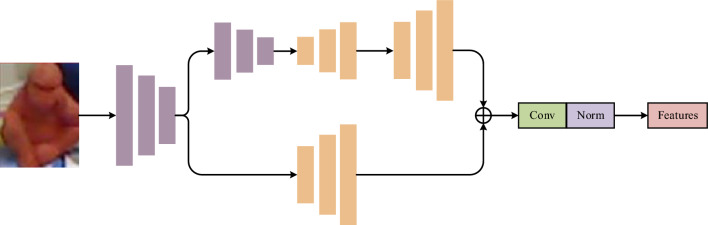
Pyramid Net structure.

Classical image recognition methods^30^, ^31^ are adequate but not sufficient in color feature extraction. Therefore, this study applies the approach introduced in^9^ to extract the RGB features. In contrast to^9^, the Pyramid Net (as shown in Fig. 5) uses simpler VGG^31^ frameworks and convolutional upsampling blocks to accommodate for the high similarity and poor resolution, reducing the number of model parameters while increasing the inference speed.

#### Geometry pipe

The coordinates of the object center point, as well as the length and breadth of the enclosing box, are included in the output of the 2D object detector, the 6D-2DH. Since depth images are aligned with the RGB images, the 6D-2DH result can be used directly on depth images, which can reduce the search space.

The method proposed in^22^, which transforms depth images into point clouds, is applied in this study. However, to improve the model's generalization ability and lessen the correlation of point features collected from comparable views, data augmentation is still required. Common transformation methods (e.g., translation, rotation, and scaling) cannot change the object shape in point clouds. In detail, the permutation invariance of point clouds^32^ does not allow them to become fundamentally different from the original data after simple data augmentation, which hinders the model enhancement effect of simple data augmentation methods.

To solve the aforementioned problem, this study proposes using minor 3D deformation, which is based on the 3D deformation^22^. Therefore, instead of allocating individual points to the nearest 3D box surface, minor 3D deformation is used to directly rotate and extend the total point cloud in small increments. Given the initial points *P*, the points generated after data augmentation *P*_*aug*_ can be calculated as follows:

2 $$ P_{aug} = \left( {R_{random} \times \left( {(P - \overline{P}) \times \alpha + T_{random} } \right)^{T} } \right)^{T} , $$

where ***R***_*random*_ and ***T***_*random*_ denote the rotation and translation matrices generated from random numbers within a certain range, respectively; *ɑ* is the zoom scale.

To acquire the predicted centroid coordinates and the extracted geometric features, the data obtained after point segmentation are transferred into the subsequent pointNet network^24^. Then, a max pooling layer is used to generate the geometric features. The translation of points is easier to estimate than the rotation of points because the centroid's displacement distance *T* substitutes the translation value of the entire object. The idea of a translational and rotational decoupling prediction is adopted, which consumes more resources to predict rotation *R* but a simpler network structure to predict translation *T*.

#### Feature fusion

After the color and geometric features are obtained, they need to be fused. Motivated by the results presented in^5^, this study adopt a fusion approach of concatenating feature in the channel dimension directly, as presented in Fig. 2. In contrast to^5^, in this study, max pooling is used instead of average pooling to generate global features because it can better depicts the notable parts of a feature map.

### ARED structure

This study aims to design an encoder–decoder structure capable of coding and decoding fused information to compute the object's rotation matrix, which is necessary for 6D pose estimation.

Although the transformer structure employed in^21,33^ is innovative in this domain, it has a large number of parameters, which is an unavoidable disadvantage for 6D pose estimation methods that must meet real-time detection requirements. However, a large number of parameters can entail a higher memory overhead and a longer training time.

**Figure 6:**
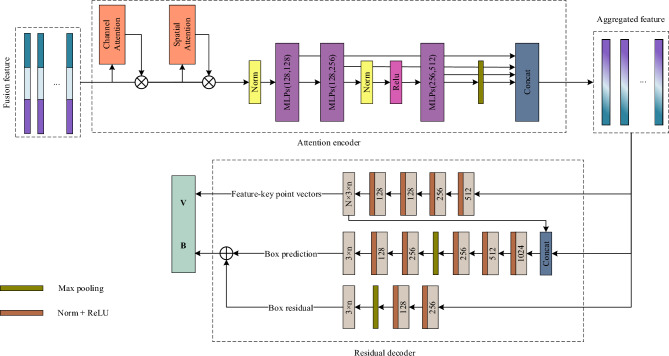
ARED structure.

This study employs the ARED structure, presented in Fig. 6, to solve the aforementioned difficulty. As shown in Fig. 6, this structure contains an encoder with an attention mechanism^34^ and a decoder with a residual structure. After fusion, the proposed network framework can fully learn features while spending as little time as possible to infer an object's 6D pose.

#### AE Structure

Recent 6D pose estimation networks^23,25,35,36^ include an essential component, namely the self-attentive mechanism^36^, which not only improves the network's learning of focus characteristics but also replaces complicated modular stacking structures. It has been demonstrated that the self-attentive mechanism is very stable and effective. This framework plays a vital role in subsequent model training, which requires long-term reliance on its outcomes.

The AE adopts a channel attention module^34^ and a spatial attention module^34^ (CASA), followed by MLPs^24,37^ to meet the encoder’s demands. The CASA in the AE structure is used to process one-dimensional features. The feature encoding is finished after processing the CASA result by a series of MLPs. It should be noted that the model is trained for the downstream task of 6D pose estimation and does not employ any specific loss terms to train the CASA.

As shown in Fig. 6, an input feature map Г_*fusion*_ has a shape of ${\mathbb{R}}^{C \times N}$, where *N* is the number of features and *C* is the number of channels for each dimensional feature before the main network. consisting of the attention encoder and residual decoder, starts. In this work, channel attention feature map Г_*channel*_ and spatial attention feature map Г_*spatial*_ are defined as follows:

3 $$ \left\{ \begin{gathered} \Gamma_{{{\text{cha}}nnel}} = \Gamma_{{{\text{fusion}}}} \times Attention_{channel} (\Gamma_{{{\text{fusion}}}} ) \hfill \\ \Gamma_{{s{\text{p}}atial}} = \Gamma_{channel} \times A{\text{tt}}ention_{spatial} (\Gamma_{channel} ) \hfill \\ \end{gathered} \right., $$

where *Attention*_*channel*_(·) is the block of the channel attention module, and *Attention*_*spatial*_(·) is the block of the spatial attention module.

Some of the subsequent blocks of the MLPs play an important role in learning the feature matrix. To obtain the final encoding result, the feature map after the max-pooling operation is concatenated with the results of the successive MLPs.

#### RD structure

Different from previous classic 6D pose estimation models^5,7,12,17^, which require an additional refinement module, recent studies^16,38–40^ have used a variety of fast approaches instead of the refinement module to increase the model's inference speed and meet the real-time requirements.

However, excluding the pose refinement operation unavoidably results in a certain accuracy loss. To address this problem, this study proposes the RD structure that has a small weight, is simple to comprehend, and has a high accuracy.

The RD employs feature-key point vectors and key point boxes to constrain the model jointly. Rotation matrices^9,16^ and quaternions^5,7^ are the most common constraints for network convergence. However, for symmetric objects, several rotation labels can correspond to the same appearance^25^, having a minimal influence on translation and contributing to mistakes in rotation estimates. In contrast, quaternions have downsides. The unit-norm limitation applies to quaternions, limiting the network`s output range^12^. Feature-key point vectors (FPVs) $V \in {\mathbb{R}}^{N \times 3 \times n}$, where *N* is the number of features, and *n* is the number of key points, estimate the rotation matrix by using vectors from each feature point to the key points^7,41^. The FPV analyzes how symmetrical objects affect the evaluation result. Similarly, the key points box (KPB),$B \in {\mathbb{R}}^{3 \times n}$, evaluates the rotation of an object based on the change of key points.

The **FPV** performs a series of convolutions, normalization, and non-linear function *Convs*(·) to evaluate vector *nV*; it computes *V* using the aggregated feature Г_*agg*_ as follows:

4 $$ V = Convs\left( {\Gamma_{agg} } \right) $$

The **KPB** contains the box prediction (BP) pipe and box residual (BR) pipe, which both include *Convs*(·) and a max pooling layer *Pooling*(·). The KPB result denotes the 3D coordinates of the *n* key points instead of a rotation matrix. The final rotation matrix $R \in {\mathbb{R}}^{3 \times 3}$ is calculated based on the key points’ positions using the Kabsch algorithm. The calculation process of the KPB is as follows:

5 $$ \left\{ \begin{gathered} B_{box} = Convs\left( {Pooling\left( {Convs\left( {\left[ {\Gamma_{agg} ,V} \right]} \right)} \right)} \right) \hfill \\ B_{residual} = Pooling\left( {Convs\left( {\Gamma_{agg} } \right)} \right) \hfill \\ B = B_{box} \oplus B_{residual} \hfill \\ \end{gathered} \right., $$

where $\oplus$ is the matrix adding operation; *B*_*box*_ and *B*_*residual*_ are the results of the BP and BR pipes, respectively.

### Loss function

This section defines the loss function employed in the proposed model. The loss function *L*_*T*_ measures the Euclidean distance between the label's centroid coordinates and the prediction's centroid coordinates, and it is defined as follows:

6 $$ \left\{ \begin{gathered} \tilde{C}_{x,y,z} = \tilde{T} - M_{seg} \hfill \\ L_{T} { = }\left\| {\left. {C_{x,y,z} - \tilde{C}_{x,y,z} } \right\|} \right._{2} \hfill \\ \end{gathered} \right., $$

where $\tilde{C}_{x,y,z}$ denotes the centroid coordinates of the label; *C*_*x,y,z*_ represents the centroid coordinates of the prediction; $\tilde{T}$ is the translation value of the label; *M*_*seg*_ is the mean of points after point segmentation.

The point vector $\tilde{V}$ from the feature point to the key point is used as a label. In addition, to constrain the rotation estimation of objects, particularly symmetric objects, *L*_*vec*_ encourages *V* to be as accurate as possible. The loss function *L*_*vec*_ of FPV is expressed as follows:

7 $$ L_{{{\text{vec}}}} = \left\| {\left. {V - \tilde{V}} \right\|_{2} } \right. $$

The essence of the KPB is to use the positions of key points to obtain the rotation matrix. The loss functions *L*_*box*_ and *L*_*residual*_ punish the errors in the positions of the predicted key points, and they are defined as follows:

8 $$ \left\{ \begin{gathered} L_{box} = \left\| {\left. {B_{box} - \tilde{B}} \right\|_{2} } \right. \hfill \\ L_{residual} = \left\| {\left. {B_{residual} - \tilde{B}} \right\|_{2} } \right. \hfill \\ \end{gathered} \right., $$

where $\tilde{B}$ denotes the key points’ coordinates for the label.

Finally, the total loss function *L*_*total*_ is defined as follows:

9 $$ L_{{{\text{total}}}} = \lambda_{1} \times L_{seg} + \lambda_{2} \times L_{T} + \lambda_{3} \times L_{vec} + \lambda_{4} \times L_{box} + \lambda_{5} \times L_{residual} , $$

where the *L*_*seg*_ represents the loss of point segmentation^24^, and the *λ*_*i*_ (*i* = 1, 2, 3, 4, 5) denotes the weight of the corresponding loss.

## Experiments

The experiments were conducted to demonstrate the proposed method’s performance in tackling the challenges that arise in two challenging datasets, namely, the LINEMOD^4^ and YCB-Video^42^ datasets, which were selected to evaluate the performance of the proposed method. The evaluation metrics included the ADD, the ADD-S, and the ADD(-S) AUC, which are all described below.

### Implementation details

The PyTorch was used to implement the proposed framework. All experiments related to model training were performed on a desktop pc with an Intel 2.40 GHz CPU and two NVIDIA 3090 GPUs, using a depth image of 640 × 480 and an RGB image of the same size; also, the 6D-2DH was used as a detector. As for the model test experiments, a device with an Intel 2.40 GHz CPU and an NVIDIA 3090 GPU was applied.

First, the 6D-2DH framework with the pretrained model^25^ was used to locate the object of interest. Then, the corresponding depth map was converted into point cloud data. The Pyramid Net was used to fine-tune the VGG. Some max-pooling layers were removed. When the fusion feature entered the attention block, the channel was unchanged, and the kernel size was set to seven. In this experiment, the units were standardized to mm. The Adam optimizer was employed to optimize the proposed network model. The initial learning rate was set to 0.001, and the learning rate decayed by 0.25 every 75 epochs. The maximum epoch number was set to 300. The weights *λ*_*i*_ (*i* = 1, 2, 3, 4, 5) were set as follows: *λ*_*1*_ = 10, *λ*_*2*_ = 0.1, *λ*_*3*_ = 10, *λ*_*4*_ = 0.01, and *λ*_*5*_ = 0.01. The ɑ value in Eq. (2) was set to 0.9; the values of *N* and *n* were set to 1000 and eight, respectively.

### Datasets

The LINEMOD^4^ is a classical dataset that has been widely used for 6D object pose estimation. Some SOTA methods^5^ ^6^ ^7^ ^11^ ^16^ ^39^ have used this dataset to construct the training and test sets, so these methods could be compared with the proposed method. Further, a Kinect camera was used to capture images, including RGB and depth images; the images were automatically aligned. This dataset contained 13 low-textured objects of different types, each of which included annotated 6D poses and object masks. The cluttered scenes, texture-less objects, and lighting variations changes denoted the main challenges in this dataset. This study employed 15% of each item sequence for model training, and the remainder was used to test the trained model.

The YCB-Video^42^ is another standard benchmark dataset, which contains 21 YCB objects of different shapes and textures. This dataset contained 92 RGB-D videos, each with a subset of the objects placed in the scene. It is challenging due to varying lighting conditions, image noise, and occlusions. In the experiment, we divided the training set and test set according to previous work^17^.

In order to train the point clouds with the process *seg* that appears in Fig. 2, we apply an automatic way^43^ to label.

### Evaluation metrics

The ADD metric defined by Eq. (10) ^44^ was used as an evaluation metric for non-symmetric objects.

10 $$ ADD{ = }\frac{1}{{\text{m}}} \times \sum\limits_{x \in P}^{{}} {\left\| {(Rx + t) - (\hat{R}x + \hat{t})} \right\|} $$

In Eq. (10), *x* denotes a total of *m* points on the object mesh *P*; *R* is the ground truth rotation; *t* is the ground truth translation; $\hat{R}$ is the estimated rotation; $\hat{t}$ is the estimated translation.

For symmetric objects, such as eggbox and glue, the ADD-S metric^44^ defined by Eq. (11) was adopted, and the average distance was calculated using the shortest distance to evaluate the model`s performance.

11 $$ ADD{ - }S{ = }\frac{1}{{\text{m}}} \times \sum\limits_{x1 \in P}^{{}} {\mathop {\min }\limits_{x2 \in P} \left\| {(R \cdot x_{1} + t) - (\hat{R} \cdot x_{2} + \hat{t})} \right\|} $$

The mean distance between two converted point sets was used as a threshold. In the evaluation process on the LINEMOD dataset, the threshold was set to 10% of the 3D object model diameter. The ADD(-S) metric, which used ADD-S for symmetrical objects and ADD for non-symmetrical objects, was adopted to compute the model's performance. In the evaluations on the YCB-Video dataset, this study followed the suggestions provided in^17^ and adopted the ADD(-S) AUC metric, which combined AUC for ADD metric used in non-symmetric objects and AUC for ADD-S metric used in symmetric objects. The ADD(-S) AUC metric denoted the area under the accuracy-threshold curve whose maximum threshold was set to 0.1 m.

### Comparison with SOTA methods

Evaluation on the LINEMOD dataset: The proposed network was compared with seven SOTA pose estimation algorithms. Based on the results in Table 1, the best mean score of the proposed method achieved in the comparison tests was 98.6%. The best scores of the other methods were as follows: PVNet^7^ (86.3%), CDPN^6^ (89.9%), HybridPose^11^ (94.5%), DenseFusion^5^ (94.3%), CloudAAE^23^ (95.5%), Crt-6d^5^ (93.5%) , CloudAAE^23^ (86.8%) without refinement, and Gao^16^ (94.6%). The proposed DON6D method performed best on 10 of the 13 objects from the LINEMOD dataset. Moreover, the proposed DON6D method outperformed the second-best method (i.e., the CloudAAE method), by 3.1%. Particularly, for the egg box object, the DON6D method achieved a performance of 100%, the same as Gao^16^. Since the most classical two-stage 6D pose estimation network, the PVNet^7^, used only RGB images as input data, while the proposed method combined the RGB images and depth images, the DON6D had a lower speed, as shown in Table 2, but improved the mean score by 12.3% compared to the PVNet. As presented in Table 2, there were differences in the speed between the proposed DON6D method and the SOTA algorithms. The DON6D with 41 FPS employed an attention residual encoder–decoder to increase speed and maintain accuracy. The results indicated that the proposed algorithm was faster than most SOTA algorithms^5,16^ ^23^ but slightly slower than the PVNet^7^.

**Table 1:** The 6D pose estimation results on the LINEMOD dataset; the ADD(-S) metric was used to compute the performance of objects; objects with “*” indicate symmetry objects; numbers written in bold denote the best results obtained in the comparison tests; methods indicated by italic letters did not include subsequent refinement.

[18]Objects	Two-stage methods					One-stage methods			
	PVNet	CDPN	HybridPose	DenseFusion	CloudAAE	*Crt-6d*	*CloudAAE*	Gao	DON6D
	^7^	^6^	^11^	^5^	^23^	^5^	^23^	^16^	(Ours)
Ape	43.6	64.4	77.6	92.3	92.5	96.4	80.2	89	**96.9**
Benchvise	**99.9**	97.8	99.6	93.2	91.8	91.3	85.7	93.1	**98.5**
Camera	86.9	91.7	95.9	94.4	88.9	84.8	61	95.9	**98.6**
Can	95.5	95.9	93.6	93.1	96.4	97.1	93.1	93.2	**98.5**
Cat	79.3	83.8	93.5	96.5	97.5	98.0	94.4	95	**99.7**
Driller	96.4	96.2	97.2	87	99	94.7	98.2	94.2	**99.4**
Duck	52.6	66.8	87	92.3	92.7	86.8	62.6	90.3	**94.8**
Egg Box*	99.2	99.7	99.6	99.8	99.8	**100**	99.8	**100**	**100**
Glue*	95.7	99.6	98.7	**100**	99	**100**	94.1	**100**	98.6
Holepuncher	81.9	85.8	92.5	92.1	93.7	92.1	84.4	92.2	**98.8**
Iron	**98.9**	97.9	98.1	97	95.9	90.1	89.5	96.5	98.5
Lamp	**99.9**	97.9	96.9	95.3	96.6	97.3	91.6	95.1	**99.9**
Phone	92.4	90.8	98.3	92.8	97.4	88	93.5	94.8	**99.1**
Average	86.3	89.9	94.5	94.3	95.5	93.5	86.8	94.6	**98.6**

**Table 2:** The speed (frames per second, FPS) of different methods on the LINEMOD dataset. Methods written in italics did not include subsequent refinement modules.

Methods	PVNet	DenseFusion	*DenseFusion*	Gao	CloudAEE	*CloudAEE*	DON6D
	^7^	^5^	^5^	^16^	^23^	^23^	(Ours)
FPS	**42**	26	34	30	22	24	41

**Figure 7:**
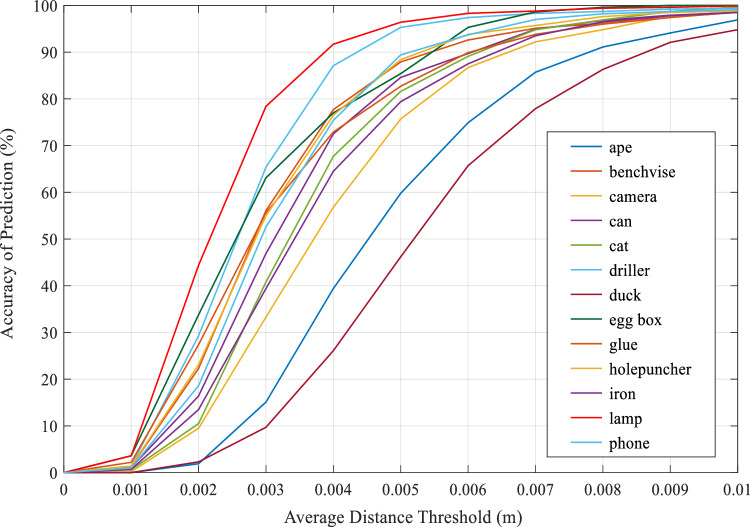
Accuracy-threshold curves for different objects from the LINEMOD dataset.

The proposed DON6D was also tested for the average distance thresholds of less than 0.01 m. This allowed for assessing how well the proposed model could perform in high-precision posture estimation tasks. As shown in Fig. 7, on the LINEMOD, the accuracy for different objects varied with the threshold. However, the curves for all types of objects were positively correlated with the mean distance threshold, with no significant anomalous parts. For all target items, except for the duck object, the accuracy exceeded 80% at a threshold of 0.007 m; at a threshold of roughly 0.072 m, the duck attained an accuracy of 80%. In addition, only the duck and the ape had an accuracy of 80% at a threshold of 0.006 m. Considering the integration of annotation and physical analysis on this dataset, there could be two causes for such results. The proposed model could detect and estimate the weakly textured, weakly illuminated objects incorrectly because their edge parts were similar to the background pixels; another factor could be a large error of the camera in capturing depth information, such as an ape, which denoted an object for which even small changes in the depth level might not be fully captured due to occlusion, shooting angle, or hardware. For example, the larger the object was (e.g., a desk lamp, iron, and vise), the smaller the average threshold required for maintaining an accurate estimation was. When the threshold was less than 0.004, the accuracy of all objects decreased sharply.

**Figure 8:**
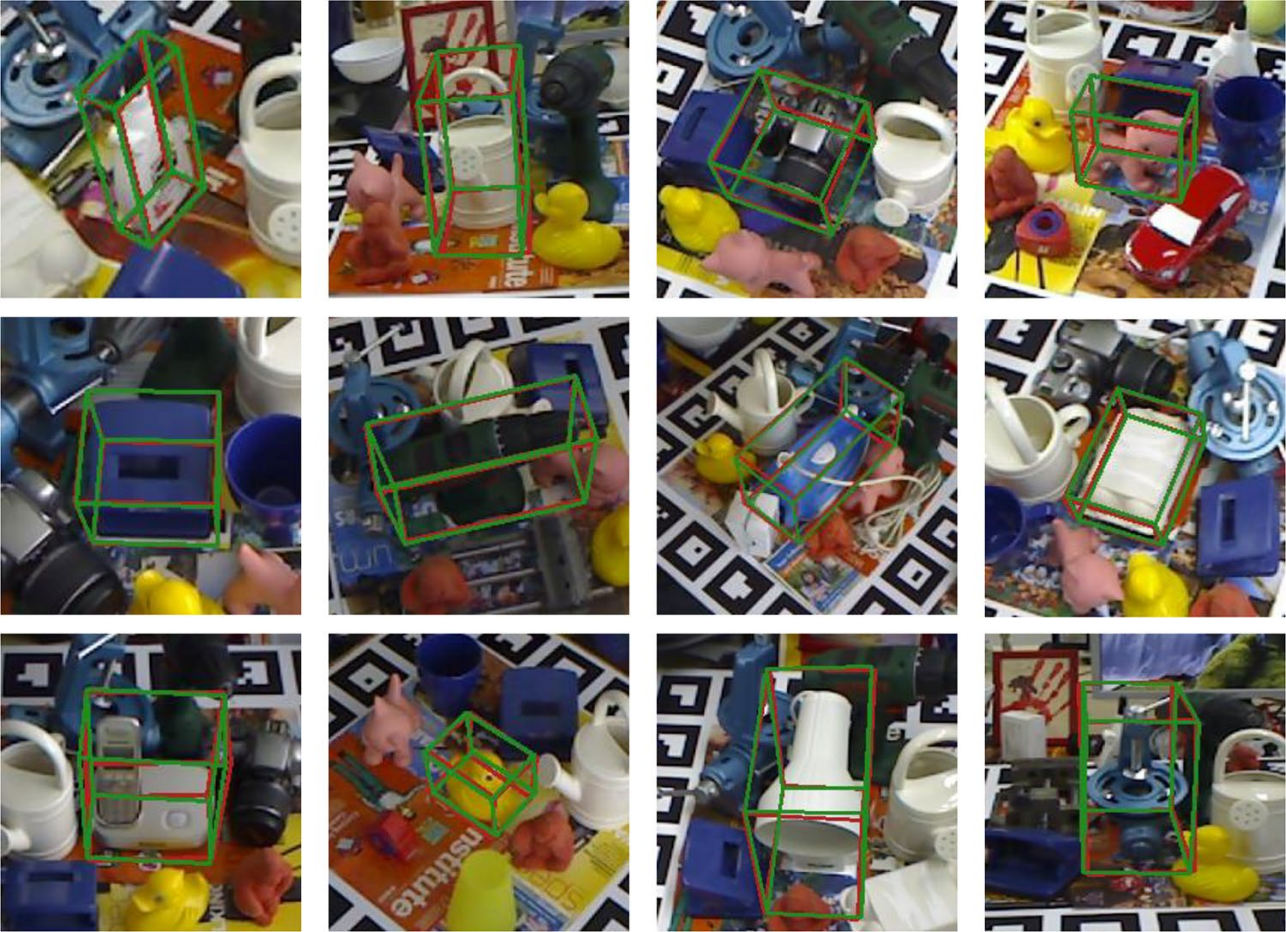
Results on the LINEMOD dataset. The green 3D bounding boxes denote the results of the proposed method; the red 3D bounding boxes represent the ground truth.

In Fig. 8, the estimation results for different types of objects obtained using different threshold values are presented.

**Figure 9:**
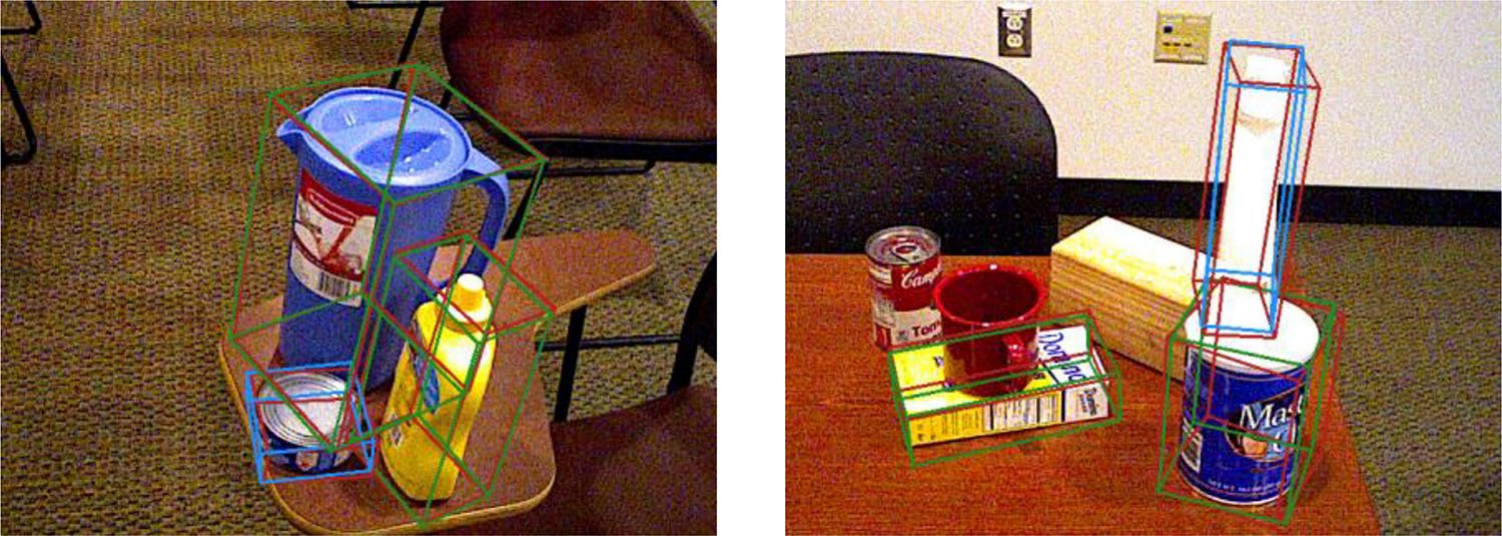
Prediction results of the proposed method on the YCB-Video dataset. The red 3D bounding boxes denote the ground truth, and the other boxes represent the estimation result.

Evaluation on the YCB-Video dataset: The results of the proposed DON6D on the YCB-Video dataset are presented in Fig. 9. Different from the LINEMOD dataset, each frame in the YCB-Video dataset included numerous objects, so there could be many occlusions and phases in the same image, posing a challenge to the proposed model. In addition, the prediction difficulty was further increased due to the inconsistency of the training and test datasets. Further, from Table 3 the proposed DON6D was compared with the SOTA algorithms^7^ ^16^ ^17^ ^39^ regarding different metrics. In terms of the ADD(-S) AUC metric, the DON6D achieved the best result of 88.3% among all methods. The DON6D outperformed the other algorithms on five objects. The proposed method performed 1.8% better than the competitive PoseCNN + ICP method^17^ in terms of the mean score, but it was more than 200 times faster than it. Particularly, the speed of the DON6D was 23 FPS, which could satisfy real-time requirements; this demonstrates the advantage of the proposed model. Compared to the recent methods, the Gao^16^ and ROPE^39^ methods, the proposed method had many high-score objects. It should be noted that the proposed method had the best performance of 2/5 on symmetric objects, outperforming the PoseCNN + ICP^17^, PVNet^7^, and ROPE^39^ methods.

**Table 3:** The 6D pose estimation accuracy on the **YCB-Video** dataset; ADD(-S) AUC metric ^17^ was used to evaluate the SOTA methods; objects with “*” denote symmetry objects.

Objects	PoseCNN + ICP	PVNet	ROPE	Gao	DON6D
	^17^	^7^	^39^	^16^	(Ours)
002_master_chef_can	69.0	81.6	71.2	67.9	**91.6**
003_cracker_box	80.7	80.5	**89.9**	89.7	80.8
004_sugar_box	**97.2**	84.9	93.2	**97.2**	95.1
005_tomato_suop_can	81.6	78.2	82.5	85.1	**87.9**
006_mustard_bottle	**97.0**	88.3	95.3	90.7	92.4
007_tuna_fish_can	83.1	62.2	88.0	79.5	**93.8**
008_pudding_box	**96.6**	85.2	90.5	89.3	79.2
009_gelatin_box	**98.2**	88.7	89.4	93.5	93.9
010_potted_meat_can	**83.8**	65.1	74.5	81.3	82.1
011_banana	**91.6**	51.8	58.8	80	86.2
019_pitcher_base	**96.7**	91.2	92.9	91	92.5
021_bleach_cleanser	**92.3**	74.8	77.4	88.3	84.7
024_bowl*	78.3	89.0	70.8	**93.1**	83.5
025_mug	81.4	81.5	89.1	83.3	**92.2**
035_power_drill	**96.9**	83.4	89.4	82.6	86.9
036_wood_block*	90.5	71.5	70.6	**91.0**	82.7
037_scissors	78.4	54.8	**84.8**	77	84.1
040_large_marker	85.4	35.8	53.3	**91.1**	85.5
051_large_clamp*	75.4	66.3	77.1	71.5	**93.5**
052_extra_large_clamp*	65.3	53.9	55.2	68.3	**91.5**
061_foam_brick*	**97.1**	80.6	83.8	95.1	94.9
Average	86.5	73.4	79.9	85.1	**88.3**

### Ablation studies

The proposed DON6D was tested under different setups on the LINEMOD dataset to explore the proposed modules’ effects on the overall model performance. Compared to the other methods used in the comparison^5^ ^7^ ^16^ ^23^, the DON6D included three innovations. First, in the AE, the channel attention mechanism and the spatial attention mechanism were used to process the fused feature maps successively so that the information of interest about the network could be better used. Then, the RD, which included the FPV, BR, and BP, was used to improve the speed while maintaining accuracy. Finally, the DDA was used to further enhance the performance of the proposed network. The ADD(-S) metric was used to evaluate the performance of the mentioned innovations of the DON6D on the LINIMOD dataset, as shown in Table 4.

**Table 4:** The results of the ablation studies conducted on the LINIMOD dataset; ”CASA” represents the channel attention and spatial attention module; ”FPV” denotes the feature-key point vector pipe; ”BR” is the box residual pipe; “DDA” indicates the dual data augmented module.

CASA	FPV	BR	DDA	Acc (%)
✗	✗	✗	✗	83.7
✓	✗	✗	✗	87.4
✓	✓	✗	✗	90.6
✓	✓	✓	✗	93.7
✓	✓	✓	✓	98.1

## Conclusion

This study introduces the DON6D model, which is a decoupled one-stage network for 6D pose estimation. The DON6D model decouples the 6D pose estimation process into the object localization, feature extraction and fusion, and attention residual encoding–decoding processes. In the object localization process, a 6D-2DH model, which is faster and lighter than object segmentation approaches, is used to locate the object`s position. Then, to enhance the generalization ability of the proposed model, the DDA is applied to feature extraction and fusion. In addition, the AE is used to replace complex modular stacking systems. Further, due to the difficulties of rotation matrix prediction and the restrictions of common constraints, the RD that combines the feature-key point vector pipe and the box residual pipe is used. The results of the experiments on publicly available datasets demonstrate that the proposed DON6D can achieve an accurate real-time estimation and outperform the SOTA pose estimate algorithms in terms of accuracy.

In the future, on the premise of maintaining accuracy, the branching of the proposed network could be reduced to make the network faster.

## Data Availability

The data used to support the findings of this article is publicly available at https://bop.felk.cvut.cz/datasets/ and https://rse-lab.cs.washington.edu/projects/posecnn/ .
